# A quality improvement project on nicotine replacement therapy in Shannon Clinic (Northern Ireland)

**DOI:** 10.1192/bjo.2021.564

**Published:** 2021-06-18

**Authors:** Eileen Moss

**Affiliations:** Shannon Clinic

## Abstract

**Aims:**

The aims of this quality improvement project were to determine if Nicotine Replacement Therapy was being prescribed correctly in Shannon Clinic in Northern Ireland and also to improve the rates of correct prescribing of Nicotine Replacement Therapy in the aforementioned unit.

**Background:**

There are several different types of Nicotine Replacement Therapy currently available. Shannon Clinic is a smoke-free clinical environment therefore patients who smoke are offered Nicotine Replacement Therapy on admission. When I was working at Shannon Clinic I became aware that there was no clear guidance available to medical staff on the wards regarding prescribing Nicotine Replacement Therapy and therefore I decided to carry out this quality improvement project.

**Method:**

An audit of drug charts was done on the patients who were under the care of the consultant that I worked with. In total nine drug charts were included in the audit. After the audit was complete, I produced a poster to show how to correctly prescribe Nicotine Replacement Therapy. A copy of this poster was placed on each ward in Shannon Clinic. After a period of time the drug charts were re-audited to see if there had been an improvement in the rates of correct prescribing of Nicotine Replacement Therapy.

**Result:**

In total, 22% of the drug charts which were included in the audit had Nicotine Replacement Therapy prescribed incorrectly on them. After the inclusion of a poster outlining how to prescribe Nicotine correctly on each ward in Shannon Clinic, 0% of drug charts had Nicotine Replacement Therapy prescribed incorrectly on them. This was an improvement of 22%.

**Conclusion:**

This quality improvement project was successful at reducing the rates of incorrect Nicotine Replacement Therapy in Shannon Clinic. In the future it is my hope that this quality improvement project should lead to the correct prescribing of Nicotine Replacement Therapy for all patients in Shannon Clinic. It should also lead to an increased awareness regarding the different types of Nicotine Replacement Therapy for medical staff working in this clinical unit.

